# Biological activities of extracts and compounds from Thai Kae-Lae (*Maclura cochinchinensis* (Lour.) Corner)

**DOI:** 10.1186/s12906-023-03979-w

**Published:** 2023-06-09

**Authors:** Lapamas Rueankham, Pawaret Panyajai, Aroonchai Saiai, Methee Rungrojsakul, Singkome Tima, Sawitree Chiampanichayakul, Kankanit Yeerong, Suvimol Somwongin, Wantida Chaiyana, Pornngarm Dejkriengkraikul, Siriporn Okonogi, Trinnakorn Katekunlaphan, Songyot Anuchapreeda

**Affiliations:** 1 Department of Medical Technology, Faculty of Associated Medical Sciences, Chiang Mai University, Chiang Mai, 50200 Thailand; 2 Department of Chemistry, Faculty of Science, Chiang Mai University, Chiang Mai, 50200 Thailand; 3 Department of Traditional Chinese Medicine, Faculty of Science, Chandrakasem, Rajabhat University, Bangkok, 10900 Thailand; 4 Center for Research and Development of Natural Products for Health, Chiang Mai University, Chiang Mai, 50200 Thailand; 5 Cancer Research Unit of Associated Medical Sciences (AMS-CRU), Chiang Mai University, Chiang Mai, 50200 Thailand; 6 Department of Pharmaceutical Sciences, Faculty of Pharmacy, Chiang Mai University, Chiang Mai, 50200 Thailand; 7 Department of Biochemistry, Faculty of Medicine, Chiang Mai University, Chiang Mai, 50200 Thailand; 8 Department of Chemistry, Faculty of Science, Chandrakasem, Rajabhat University, Bangkok, 10900 Thailand

**Keywords:** *Maclura cochinchinensis* (Lour.) Corner, Fractional extract, Resveratrol, Antioxidant, Cancer prevention, Anti-inflammation, Anti-leukaemia

## Abstract

**Background and aims:**

The purpose of this study was to investigate the biological properties of Kae-Lae (*Maclura cochinchinensis* (Lour.) Corner), a traditional medicinal plant used in Ayurvedic recipes in Thailand. To achieve this objective, heartwood samples were collected from 12 sources across Thailand. Fractional extracts (*n*-hexane, ethyl acetate, and ethanol) and the dominant compounds (morin, resveratrol, and quercetin) were examined for their abilities on cytotoxicity, antioxidant, anti-inflammation, and antileukaemic activity (Wilms’ tumour 1 protein was used as a well-known biomarker for leukaemic cell proliferation).

**Methods:**

The study used MTT to assess cytotoxicity in leukaemic cells (K562, EoL-1, and KG-1a). Antioxidant activities were evaluated using ABTS, DPPH, and FRAP assays. The anti-inflammatory activity was investigated by detecting IL-2, TNF-α, and NO using appropriate detection kits. Wilms’ tumour 1 protein expression was measured by Western blotting to determine the anti-leukaemic activity. The inhibition of cell migration was also analyzed to confirm anticancer progression.

**Results:**

Among the tested extract fraction, ethyl acetate No. 001 displayed strong cytotoxicity specifically in EoL-1 cells, while *n*-hexane No. 008 demonstrated this effect in three cell lines. Resveratrol, on the other hand, displayed cytotoxicity in all the tested cells. Additionally, the three major compounds, morin, resveratrol, and quercetin, exhibited significant antioxidant and anti-inflammatory properties. In particular, resveratrol demonstrated a noteworthy decreased Wilms’ tumour 1 protein expression and a reduction in cell proliferation across all cells. Moreover, ethyl acetate No. 001, morin, and resveratrol effectively inhibited MCF-7 cell migration. None of these compounds showed any impact on red blood cell haemolysis.

**Conclusion:**

Based on these findings, it can be concluded that Kae-Lae has promising chemotherapeutic potential against leukaemic cells, with fractional extracts (ethyl acetate and *n*-hexane) and resveratrol exhibiting the most potent cytotoxic, antioxidant, anti-inflammatory, and anti-cell migration activities.

**Supplementary Information:**

The online version contains supplementary material available at 10.1186/s12906-023-03979-w.

## Introduction

*Maclura cochinchinensis* (Lour.) Corner, commonly known as cockspur thorn or “Kae Lae” in Thai, is a scrambling shrub belonging to the Moraceae family. The heartwood of this plant has been traditionally used in Thai traditional medicine to treat chronic fever, skin infections, diarrhea, and lymph node abnormalities. The heartwood of *M. cochinchinensis* (Lour.) Corner also exhibits various biological effects, including antioxidant and antibacterial activities [1, 2]. Previous studies have shown that the *n*-hexane and chloroform extracts of *M. cochinchinensis* (Lour.) Corner have antioxidant activities. The heartwood of this plant contains various chemical constituents such as morin, resveratrol, and quercetin, among which morin (2’,3,4’,5,7-pentahydroxyflavonone) is the predominant compound [2]. The active compounds of Kae-Lae have been extensively studied for their biological activities. The current study aims to compare the cytotoxicity of fractional extracts from 12 sources of Kae-Lae across 9 provinces in Thailand. It is well known that the sources of plant materials is important for active compound productions, and variations in percentage of active compounds or percentage yields may be attributed to soil and agro-climatic conditions [3]. In addition, the study examines the abilities of possible active compounds on cytotoxicity, antioxidant, anti-inflammatory, and antileukemic activity, and compares them to the effective fractional extract. The hypothesis is that a compound with high antioxidant and anti-inflammatory activities may show high anticancer activity. It is important to note that crude extracts and active compounds should be safe for normal cells, including normal white blood cells (peripheral blood mononuclear cells or PBMCs) and red blood cells (RBCs). Many plant species have shown a variety of biological activities, including anti-inflammatory, antioxidant, and anticancer properties. For example, *Papaver somniferum* L. (*Papaver* plant) is a rich source of alkaloid and phenolic compounds and has exhibited pharmacological properties such as anticancer, antioxidant, antimicrobial, and antidiabetic activities [4]. *Crocus sativus* L. (saffron) has also demonstrated a variety of pharmacological activities such as antimicrobial, antioxidant, cytotoxic, cardioprotective, neuroprotective, antidepressant, hypolipidemic, and antihyperglycemic properties [5]. Another example is *Tradescantia*, a genus of herbaceous plants that contain phytochemical compounds such as coumarins, alkaloids, saponins, flavonoids, phenolics, terpenoids, etc. These compounds have shown antioxidant, cytotoxic, anti-inflammatory, and anticancer properties [6].

Inflammation is a crucial process for repairing tissue damage and fighting against pathogens. However, if inflammation becomes uncontrolled, it can lead to chronic inflammation, which can promote the development of cancer cells and carcinogenesis. Several pro-inflammatory cytokines, such as TNF-α, IFN-γ, IL-1, IL-2, IL-6, and IL-12, have been identified to participate in both initiation and progression of cancer. Additionally, pro-inflammatory cytokines can induce the generation of free radicals, including inducible nitric oxide synthase (iNOS) and reactive oxygen species (ROS), during chronic inflammation. These free radicals can damage DNA and cause mutations, leading to tumor initiation [7]. Therefore, inflammation plays a crucial role in the initiation, progression, and prognosis of cancer. It is important to prevent uncontrolled inflammation as a means of cancer prevention.

In this study, we investigated the potential antioxidant, anti-inflammatory, and anticancer activities of Kae-Lae. Specifically, we evaluated the anti-leukaemic stem cell activity of Kae-Lae using KG-1a cell line as a model for anticancer activity. Leukaemic stem cells (LSCs) are abnormal haematopoietic cells that are characterized by the CD34^+^/CD38^−^ protein surface marker phenotype [8–10]. These cells share many characteristics with hematopoietic stem cells and are notoriously difficult to eliminate [11]. Moreover, LSCs are typically more resistant to chemotherapeutic agents than normal leukaemic cells, which contributes to chemoresistance and relapse [12]. LSCs also express many leukaemia-related proteins and genes, which promote quiescence, self-renewal, and differentiation into leukaemic blasts [13, 14]. In our study, we focused on Wilms’ tumour 1 (WT1) protein, which is commonly overexpress in KG-1a leukaemic stem cells [15, 16]. This protein is a well-known biological marker involved in leukaemic cell proliferation and is highly expressed in in acute myeloblastic leukaemia (AML) and acute lymphoblastic leukaemia (ALL) [17–21]. Additionally, overexpression of WT1 mRNA is also found in chronic myelocytic leukaemia (CML) and myelodysplastic syndrome, and is associated with disease progression [22–26]. To determine the anti-leukaemic stem cell activity of Kae-Lae, we evaluated its ability to suppress WT1 protein expression, which has not been previously reported for the compounds found in Kae-Lae (i.e., morin, resveratrol, and quercetin).

Previously, morin has been studied for its antioxidant, anti-inflammatory, and antidiabetic properties, as well as its activity to induce apoptosis in U937 cells [27, 28] and arrest cell cycle and induce apoptosis in HL-60 cells [29]. Resveratrol, on the other hand, has been shown to have various pharmacological effects, including antioxidant, anti-aging, anti-obesity, anti-diabetes, cardioprotection, and neuroprotection properties [30]. It has also been found to inhibit the growth of AML, ALL, and CML leukaemic cells by inducing apoptosis, autophagy, and cell cycle arrest [31], and to enhance the effects of As_2_O_3_ in inhibiting primitive AML and/or CML progenitor cell growth with apoptosis and autophagy [32]. Similarly, quercetin has antioxidant, anti-inflammatory, and anticancer activities [33]. And has been found to induce cell death in AML cells via downregulation of VEGF/Akt signaling pathways and mitochondria-mediated apoptosis [34]. It also causes G2/M phase cell cycle arrests in multiple myeloma cell lines [35], and causes apoptosis induction in KG1a cells by a synergistic effect with TRAIL [36]. However, the anti-leukaemic stem cell activity and the underlying mechanism of Kae-Lae have not been reported. This study aims to investigate the anti-leukemic stem cell activity of the fractional extracts of Kae-Lae and its dominant compounds against KG1a cells compared with human eosinophilic leukemia EoL-1 cells and human myelocytic leukemia K562 cells in vitro.

## Materials and methods

### Plant materials

Wild Kae-Lae plants were collected from 12 sources in Thailand including Bangkok, Nonthaburi, Lopburi, Phitsanulok, Phuket, Chiang Mai, Udon Thani, Roi Et, and Chiang Rai provinces during April 2020. The dried Kae-Lae materials were ground to produce fine powder with a mixer blender.

### Chemical materials

Morin hydrate and resveratrol were purchased from Tokyo Chemical Industry (TCI) (Tokyo, Japan). Quercetin, Folin–Ciocalteu solution, 2,20-azino-bis-3-ethylbenzthiazoline-6-sulphonic acid (ABTS), 1,1-diphenyl-2-picrylhydrazyl (DPPH), and 3-(4,5-Dimethylthiazol-2-yl)-2,5-diphenyltetrazolium bromide (MTT) were purchased from Sigma-Aldrich (St. Louis, MO, USA). Ferric chloride, ferrous sulfate, sodium carbonate, sodium acetate, aluminum trichloride, and potassium persulfate were purchased from Merck (Darmstadt, Germany). Dulbecco’s modified Eagle’s medium (DMEM), RPMI-1640 medium, and penicillin–streptomycin were purchased from GIBCO Invitrogen™ (Grand Island, NY, USA). Fetal bovine serum (FBS) was obtained from Biochrom AG (Berlin, Germany). Protein markers, 0.5 M Tris–HCl, pH 6.8 solution, 1 M Tris–HCl, pH 8 solution, and 30% acrylamide/bis solution, were purchased from Bio-Rad Laboratories (Richmond, CA, USA). Ethanol, *n*-hexane, ethyl acetate, and dimethyl sulfoxide (DMSO) were purchased from Labscan (Dublin, Ireland). HPLC grade ethanol was purchased from Carlo Erba Reagents (Cornaredo, Italy).

### Plant extraction

The fractions of Kae-Lae plants (12 sources) were extracted using *n-*hexane (Hex), ethyl acetate (EtOAc), and ethanol (EtOH). These three organic solvents were used to extract due to difference of polarities. The relative polarity of Hex, EtOAc, and EtOH was 0.009, 0.228, and 0.654, respectively when compared with water (1.000). Thus, three compound groups (non-polar, moderate polar, and polar compounds) were collected for our experiments.

The air-dried powdered heartwood (100 g) of Kae-Lae plants (12 sources) were extracted with Hex (500 mL × 3 times), EtOAc (500 mL × 3 times), and EtOH (500 mL × 3 times). The Hex, EtOAC, and EtOH extracts were filtered and then concentrated under reduced pressure, to obtain 36 extracts. The crude fractional extracts were evaluated for antioxidant, anticancer, and cytotoxic activities.

### Determination of morin, resveratrol, and quercetin contents by high performance liquid chromatography (HPLC)

EtOAc No. 001 was analysed for morin, resveratrol, and quercetin using Shimadzu HPLC Prominence-i LC-2030C 3D Plus, equipped with the variable wavelength detectors (VWD) (Shimadzu Corporation, Kyoto, Japan). Reverse phase column, Zorbax SB − C18 column (4.6 × 150 mm, 5 μm) with C18 guard column (Agilent Technologies, Inc., Santa Clara, CA, USA) was applied as a stationary phase. Morin was analysed using mobile phase condition for morin in isocratic solvent system; 0.5% acetic acid water and acetronitrile (80:20, *v/v*). Morin was detected at the wavelength of 355 nm [37]. Resveratrol was detected at the wavelength of 320 nm. The temperature of the column was set at 30 °C. The mobile phase consisted of acetronitrile (eluent A) and 1% formic acid in water (eluent B). Quercetin analysis was performed at a column temperature of 25 °C and detected at the wavelength of 366 nm. A mobile phase used the gradient solvent system with methanol (eluent A) and 2% acetic acid in water (eluent B).

Hex No. 008 was analysed using Agilent 1200 series HPLC system, equipped with the Agilent 1200 infinity series variable wavelength detectors (VWD) (Agilent Technologies, Inc., Santa Clara, CA, USA). A stationary phase for morin used a reverse phase column, Hypersil™ ODS C18 column (4.6 × 100 mm, 3.5 μm) with C18 guard column (Thermo Fisher Scientific, MA, USA). The temperature of the column was set at 25 °C. The mobile phase using isocratic solvent system consisted of 0.5% acetic acid water and acetronitrile (80:20, *v/v*). Morins were detected at the wavelength of 355 nm [37]. Resveratrol was detected at the wavelength of 320 nm with a stationary phase, Poroshell 120-EC-C18 (4.6 × 150 mm, 5.0 µM) (Agilent Technologies, Inc., Santa Clara, CA, USA). The temperature of the column was set at 30 °C. A mobile phase consisted of acetronitrile (eluent A) and 1% formic acid in water (eluent B). Quercetin was analysed using Poroshell 120-EC-C18 (4.6 × 150 mm, 5.0 µM), with temperature set at 25 °C and detected at the wavelength of 366 nm. A mobile phase was the gradient solvent system with methanol (eluent A) and 2% acetic acid in water (eluent B).

These compounds in the crude fractional extracts were identified according to the HPLC retention time (RT) and UV absorbance, in comparison with commercial standards.

### Cell culture

Leukaemic cell lines including KG-1a (leukaemic stem cell-like cell line with stem cell population), EoL-1 (human promyelocytic leukaemic cell line), and K562 (human erythroid leukaemic cell line) were cultured as previously described [38, 39].

### Cytotoxic determination by MTT assay

The MTT (3-(4,5-dimethylthiazol-2-yl)-2,5-diphenyltetrazolium bromide) assay was used to determine the cytotoxicity of Kae-Lae extracts and active compounds on leukaemic cells. The method was described previously [40]. K562 (1.0 × 10^5^ cells/mL), EoL-1 (3.0 × 10^5^ cells/mL), and KG-1a (2.0 × 10^5^ cells/mL) were added samples and then incubated for 48 h. The percentage of surviving cells was calculated using the following equation:

1 $$\%\;\mathrm{Cell}\;\mathrm{viability}\;=\frac{\mathrm{Mean}\;\mathrm{absorbance}\;\mathrm{in}\;\mathrm{test}\;\mathrm{well}}{\mathrm{Mean}\;\mathrm{absorbance}\;\mathrm{in}\;\mathrm{vehicle}\;\mathrm{control}\;\mathrm{well}}\;\times100$$

The 50% inhibitory concentration (IC_50_) was determined as the lowest concentration that inhibited cell growth by 50% compared with the untreated control.

### Trypan blue exclusion test

According to the principle of trypan blue exclusion, viable cells with intact membranes can exclude trypan blue dye, while dead cells with compromised membranes are stained by the trypan blue dye solution. A cell suspension and trypan blue dye solution (0.2%) were mixed, and viable and dead cells were counted under microscope using a hemacytometer [40]. Then, the percentage of viable cells was calculated.

### Cytotoxicity of PBMCs

Method was done as previously described [40]. Briefly, peripheral blood mononuclear cells (PBMCs) were obtained from healthy donors using Ficoll-Hypaque density-gradient centrifugation using Lymphoprep™ solution (Axis-Shield, Oslo, Norway). The PBMCs (1 × 10^6^ cells/mL) were cultured in complete RPMI-1640 medium in 96-well plates overnight in a CO_2_ incubator. Various concentrations (3.125 − 100 µg/mL) of crude fractional extracts and compounds (morin, resveratrol, and quercetin) were added. Then cells were further incubated for 48 h. The survival rate of cells was assessed using the MTT assay.

### Antioxidant activity

Crude fractional extracts and their biological active compounds (morin, resveratrol, and quercetin) were investigated for antioxidant activities using 2,2'-azinobis 3-ethylbenzothiazoline-6-sulphonate (ABTS) assay, 2,2’‐diphenyl‐1‐picrylhydrazyl‐hydrate (DPPH) assay, and ferric reducing antioxidant powder (FRAP) assay.

### ABTS assay

The ABTS ^•^^+^ scavenging activity of crude fractional extracts and their biological active compounds were investigated using ABTS assay [41]. Briefly, the sample (20 μL) was mixed with of the ABTS^•^^+^ solution (180 μL) that had been prepared overnight. The combined mixture was incubated at room temperature (25 °C) for 5 min before assessing the UV absorbance at 750 nm with a microplate reader (Spectrostar Nano, BMG Labtech GmbH, Ortenberg, Germany). The findings were described as Trolox equivalent antioxidant capacity (TEAC). All experiments were conducted in triplicate.

### DPPH assay

The DPPH^•^ scavenging activity of crude fractional extracts and their biological active compounds were investigated using DPPH assay [41]. Briefly, the sample (20 μL) was mixed with 167 μM DPPH solution (180 μL). The combined mixture was incubated at room temperature (25 °C) in the dark for 30 min before assessing the UV absorbance at 520 nm with a microplate reader (Beckman CoulterDTX880, Fullerton, CA, USA). The DPPH^•^ scavenging activity was presented in terms of the percentage of inhibition that was calculated using the following equation:

2 $$\%\;\mathrm{DPPH}^\cdot\;\mathrm{inhibition}\;=\frac{\mathrm A-\mathrm B}{\mathrm A}\;\times100$$

A is the UV absorbance of the mixture in the absence of a sample solution, and B is the UV absorbance of the mixture in the absence of a sample solution. L-ascorbic acid served as a positive control. All experiments were conducted in triplicate.

### FRAP assay

The ferric reducing antioxidant power of crude fractional extracts and their biological active compounds were evaluated using FRAP assay [41]. Briefly, sample (20 μL) was mixed with FRAP solution (180 μL). The combined mixture was incubated at room temperature (25 °C) in the dark for 5 min before assessing the UV absorbance was measured at 595 nm with a microplate reader (Beckman CoulterDTX880, Fullerton, CA, USA). The findings were described as equivalent concentration (EC_1_). L-ascorbic acid served as a positive control. All experiments were conducted in triplicate.

### Anti-inflammatory activity

Anti-inflammatory activities of fractional extracts and active compounds (morin, resveratrol, and quercetin) were investigated employing inhibitory activities against nitric oxide (NO) and cytokine (IL-2 and TNF-α) secretions [42]. Mouse monocyte macrophage RAW 264.7 cells (American Type Culture Collection, ATCCTIB-71) were stimulated with lipopolysaccharide (LPS) or phytohemagglutinin (PHA). The LPS treated RAW 264.7 cells served as a vehicle control, of which the NO production, and the secreted cytokines were defined as 100%, whereas a negative control was non-treated RAW 264.7 cells. Dexamethasone (an anti-inflammatory drug) was used as a positive control.

### Nitric Oxide (NO) measurement

Briefly, 1 × 10^5^ cells/well in DMEM were seeded and incubated for 24 h in a CO_2_ incubator set at 37 °C and 5% CO_2_/95% humidified air. Then cells were treated with Kae-Lae extracts or purified compounds. Following 2-h of incubation in a CO_2_ incubator, LPS was added to make a final concentration of 1 μg/mL. After 24 h in a CO_2_ incubator, the media were removed and centrifuged at 13,500 × g for 10 min. The supernatant was analysed for the level of NO using Total Nitric Oxide Assay kit, according to the manufacturer’s protocol (Invitrogen™, ThermoFisher Scientific, MA, USA). The optical density was measured at 540 nm using a multimode detector (Beckman CoulterDTX880, Fullerton, CA, USA).

RAW 264.7 cell viability was also determined with the assay kit using MTT assay. The supernatant was removed, and MTT was added. After 2 h in a CO_2_ incubator, the supernatant was removed, and the formazan crystal were dissolved with DMSO. The optical density was measured at 578 nm and corrected with the reference wavelength of 630 nm using a multimode detector (Beckman CoulterDTX880, Fullerton, CA, USA). NO inhibitions were calculated using the following equation:

3 $$\%\;\mathrm{NO}\;\mathrm{inhibition}\;=\frac{\mathrm A\;-\;\mathrm B}{\mathrm A}\;\times100$$

A is the optical density of the mixture without the sample, while B is the optical density of the mixture with the sample. Dexamethasone was used as a positive control. All experiments were conducted in triplicate.

### IL-2 and TNF-α measurement

PBMCs (1 × 10^5^ cells/well in RPMI-1640 for IL-2) and Raw cells (1 × 10^5^ cells/well in DMEM for TNF-**α**) were seeded for 24 h in a CO_2_ incubator set at 37 °C and 5% CO_2_/95% humidified air. Then cells were treated with Kae-Lae fractional extracts (IC_20_ value) or pure compounds (IC_20_ value), or dexamethasone (1 μg/mL) were added. Following 2-h of incubation in a CO_2_ incubator, LPS was treated for a final dose of 1 μg/mL under conditions of TNF-α measurement. In contrast, PHA was added to induce inflammation in IL-2 measurement. After 24 h, the media were removed and centrifuged at 13,500 × g for 10 min and 100 μL of the supernatant was analysed by the protocol of R&D Systems, Minneapolis, MN, USA using ELISA technique. The absorbance was measured at 450 nm and corrected with the reference wavelength of 570 nm (Beckman CoulterDTX880, Fullerton, CA, USA).

Viability of PBMCs and RAW 264.7 cells was also determined using MTT assay as described previously [40]. IL-2 and TNF-α secretion inhibitions were calculated as bellow:

4 $$\%\,\mathrm{Cytokine}\,\mathrm{inhibition}=\frac{\text{A-B}}{\text{A}}\,\times{100}$$

A is the optical density of the mixture without the sample, while B is the optical density of the mixture with the sample. Dexamethasone was a positive control. All experiments were done in triplicate.

### RBC haemolysis induction

Method was done as previously described [40]. The whole blood samples were obtained from normal volunteers. Red blood cells were separated by centrifugation and washed 2 times with 0.9% NaCl. Then, 2% RBC suspension was incubated with crude fractional extracts and compounds at 37 °C for 30 min. Triton-X 100 (2%) and NaCl (0.9%) were positive and negative controls, respectively. Then, the supernatant was collected, and haemoglobin concentration was measured by spectrophotometry at 540 nm. The haemolysis induction was calculated using the following equation:

5 $$\%\;\mathrm{Haemolysis}\;\mathrm{induction}=\frac{\text{Absorbance of sample}}{\text{Absorbance of positive contr}\text{ol}}\,\times{100}$$

### Western blot analysis

Leukaemic stem cells (KG-1a) and leukaemic cells (EoL-1 and K562) were used as cell models which overexpressed WT1 protein expression. Cells were treated with crude fractional extracts and active compounds at a 20% growth inhibition (IC_20_ value) of each extract. Following 48-h of incubation, cells were washed. The whole protein was extracted using RIPA buffer. The protein concentration was determined by the Pierce™ BCA Protein Assay Kit (ThermoFisher Scientific, IL, USA). Extracted protein sample at 30 − 50 μg was loaded to 12% SDS-PAGE and then transferred to PVDF membranes. The method was done as previously described [40]. The specific protein bands were determined by Luminata™ Forte Western HRP substrate (Merck, Darmstadt, Germany) and exposed to X-ray film (Sakura, Japan). Quantity One 1-D Analysis software (Bio-Rad, USA) was used to quantify densitometry. The density values of WT1 bands were normalized to GAPDH bands.

### Rate of proliferation

KG-1a, EoL-1 and K562 cells were cultured with crude fractional extracts and active pure compounds at various concentrations (10 − 20% growth inhibition; IC_10_ − IC_20_ values) of each extract for 48 h, at a 20% growth inhibition (IC_20_ value) of each extract for 24 and 48 h. Following incubation, cells were harvested, and the total cell number was counted using trypan blue exclusion method. Three independent experiments were plotted as a dose–response curve and compared with the vehicle control. Meanwhile, the total cell number of each cell line after fractional extract and pure compound treatments at 24 and 48 h were calculated and plotted as a time-response curve. Slopes from each graph were considered as the rate of cell proliferation.

### Cell migration assay

Inhibitory effect of the extracts on cell migration of MCF-7 cells was assessed by wound healing scratch assays. First, cells were plated into 24-well plates and incubated in a CO_2_ incubator with humidified atmosphere of 5% CO_2_. After reaching the confluent monolayer forms, a longitudinal wound was created by scratching using SPL™ Scar Scratcher. Then, the non-toxic dose of each extract in complete DMEM medium was added into each well and cell migration was monitored and observed under Leica DM IL LED inverted microscopy and the images of cell-free area were captured at 0, 6, 12, and 24 h after wounding, then measured using ImageJ software. The percentage of wound width was calculated by the following formula:

6 $$Wound\;width\;(\%)\;=\;(Cell-free\;areax.h\;\times\;100)/\;Cell-free\;area0.h$$

Where “Cell-free area_x.h_” is the cell-free area at any time point of imaging while “Cell-free area_0.h_” is the cell-free area at the beginning of wounding.

### Statistical analysis

Data are presented as the mean ± standard deviation (SD) or the mean ± standard error of the mean (SEM) from three independent experiments. The statistical differences between the means were analysed by student t-test and one-way ANOVA. The differences were considered significant when the probability value obtained was found to be less than 0.05 (*p* < 0.05).

## Results

### Yield of extracts

Table 1 shows the percentage yield of crude fractional extracts obtained from different regions of Thailand. EtOH fractional extract showed the highest yield when compared with Hex and EtOAc fractional extracts. Furthermore, EtOAc fractional extract had higher yield than Hex fractional extract.

**Table 1:** Percentage yield of crude fractional extracts obtained from Kae-Lae

Kae-Lae (Source)	Yield (% w/w)		
	Hex	EtOAc	EtOH
001 (Bangkok 1)	0.42 ± 0.02	3.33 ± 0.10	8.35 ± 0.09
002 (Bangkok 2)	0.28 ± 0.02	2.85 ± 0.08	6.38 ± 0.10
003 (Bangkok 3)	0.33 ± 0.02	2.57 ± 0.09	7.69 ± 0.09
004 (Bangkok 4)	0.31 ± 0.01	3.32 ± 0.09	7.50 ± 0.10
005 (Nonthaburi)	0.27 ± 0.02	2.63 ± 0.06	6.45 ± 0.07
006 (Phitsanulok)	0.26 ± 0.01	3.06 ± 0.08	6.62 ± 0.12
007 (Lopburi)	0.28 ± 0.01	2.82 ± 0.09	9.56 ± 0.13
008 (Phuket)	0.64 ± 0.02	2.45 ± 0.06	5.18 ± 0.09
009 (Chiang Mai)	0.27 ± 0.01	3.24 ± 0.05	7.67 ± 0.10
010 (Udon Thani)	0.54 ± 0.01	3.22 ± 0.07	5.81 ± 0.11
011 (Roi Et)	0.33 ± 0.02	3.41 ± 0.07	6.82 ± 0.07
012 (Chiang Rai)	0.56 ± 0.01	3.15 ± 0.06	5.35 ± 0.07

Data present mean ± SD

### Cytotoxicity screening of crude fractional extracts from Kae-Lae in KG-1a, EoL-1, K562, and PBMCs by MTT assay

Crude fractional extracts from Kae Lae (Hex, EtOAc, and EtOH) were examined for their cytotoxicity in leukaemic stem cells (KG-1a) and leukaemic cells (K562 and EoL-1) by MTT assay. Cytotoxicity of crude fractional extracts in KG-1a cells showed that Hex Nos. 008 (from Phuket), 010 (from Udon Thani), and 012 (from Chiang Rai) had good cytotoxicity in KG1a cells with IC_50_ values of 55.21 ± 4.85, 83.44 ± 5.91, and 66.98 ± 1.62 µg/mL, respectively (Table 2). Therefore, these extracts were selected for further investigation. These candidate Kae-Lae extracts were investigated for their cytotoxicity in PBMCs. Hex Nos. 008, 010, and 012 showed low toxicities in PBMCs at concentrations of 3.125 − 12.5 µg/mL and increased their cytotoxicity at higher concentrations (Table 2).

**Table 2:** IC_50_ values (μg/mL) of crude fractional extracts from 12 sources of Kae-Lae in K562, KG-1a, EoL-1, and PBMCs

Fractional extracts (Provinces)	IC_50_ values (mean ± SD, μg/mL)			
	K562	EoL-1	KG-1a	PBMCs
001 (Bangkok 1)				
Hex	> 100	84.30 ± 3.68	> 100	ND
EtOAc	> 100	10.20 ± 2.08^a^	> 100	> 100
EtOH	> 100	33.46 ± 2.47	> 100	ND
002 (Bangkok 2)				
Hex	> 100	56.57 ± 8.23	> 100	ND
EtOAc	> 100	19.84 ± 1.73	> 100	ND
EtOH	> 100	47.24 ± 1.16	> 100	ND
003 (Bangkok 3)				
Hex	> 100	> 100	> 100	ND
EtOAc	> 100	21.77 ± 1.04	> 100	ND
EtOH	> 100	44.89 ± 1.54	> 100	ND
004 (Bangkok 4)				
Hex	> 100	> 100	> 100	ND
EtOAc	> 100	24.76 ± 3.15	> 100	ND
EtOH	> 100	43.47 ± 2.41	> 100	ND
005 (Nonthaburi)				
Hex	> 100	> 100	> 100	ND
EtOAc	> 100	23.73 ± 2.42	> 100	ND
EtOH	> 100	43.15 ± 2.15	> 100	ND
006 (Phitsanulok)				
Hex	> 100	> 100	> 100	ND
EtOAc	> 100	23.74 ± 2.88	> 100	ND
EtOH	> 100	41.27 ± 2.38	> 100	ND
007 (Lopburi)				
Hex	> 100	> 100	> 100	ND
EtOAc	> 100	21.15 ± 1.69	> 100	ND
EtOH	> 100	47.40 ± 4.70	> 100	ND
008 (Phuket)				
Hex	56.58 ± 5.93	12.21 ± 1.72^a^	55.21 ± 4.85	37.73 ± 3.93
EtOAc	> 100	20.62 ± 3.51	> 100	ND
EtOH	> 100	> 100	> 100	ND
009 (Chiang Mai)				
Hex	> 100	51.53 ± 5.21	> 100	ND
EtOAc	> 100	19.26 ± 2.81	> 100	ND
EtOH	> 100	75.61 ± 5.00	> 100	ND
010 (Udon Thani)				
Hex	73.14 ± 2.33	13.63 ± 1.41	83.44 ± 5.91	41.41 ± 7.75
EtOAc	> 100	24.07 ± 0.21	> 100	ND
EtOH	> 100	39.67 ± 1.13	> 100	ND
011 (Roi Et)				
Hex	> 100	46.02 ± 0.27	> 100	ND
EtOAc	> 100	22.66 ± 0.72	> 100	ND
EtOH	> 100	35.18 ± 0.21	> 100	ND
012 (Chiang Rai)				
Hex	56.49 ± 1.17	12.16 ± 2.23^a^	66.98 ± 1.62	40.91 ± 4.17
EtOAc	> 100	25.43 ± 3.27	97.36 ± 0.04	ND
EtOH	> 100	> 100	> 100	ND

*ND* not determined

^a^Good cytotoxicity

The cytotoxicity of crude fractional extracts from Kae-Lae were studied in leukaemic EoL-1 cells. The cytotoxicity of Hex Nos. 003 − 007 had no toxicities in EoL-1 cells. However, Hex Nos. 008, 010, and 012 had high cytotoxicity in EoL-1 cells (Table 2). Hex Nos. 008 and 012 showed no significant differences of cytotoxicity. Thus, Hex No. 008 was selected for further study due to its higher yield. Furthermore, Hex Nos. 001, 002, 009, and 011 also showed cytotoxicity in EoL-1 cells. Likewise, these extracts also had more toxicity in EoL-1 cells than the normal PBMCs. Interestingly, EtOAc No. 001 (from Bangkok 1) showed the highest cytotoxicity, especially in EoL-1 cells with an IC_50_ value of 10.20 ± 2.08 µg/mL. However, it did not show significant difference from Hex No. 008 (12.21 ± 1.72 µg/mL). EtOAc Nos. 002 − 012 had cytotoxicity in EoL-1 cells with the IC_50_ values ranging from 19.84 − 25.43 µg/mL. Almost EtOH extracts (10 samples) from Kae-Lae had cytotoxicity in EoL-1 cells with IC_50_ values during 33 − 75 µg/mL. However, all EtOH extracts showed lower cytotoxicity in EoL-1 cells than those of Hex and EtOAc fractional extracts.

In K562 cells, Hex Nos. 008, 010, and 012 showed cytotoxicity with IC_50_ values ranging from 56.49 − 73.14 µg/mL (Table 2).

### Morin, resveratrol, and quercetin contents of Kae-Lae fractional extract by HPLC determination

**Fig. 1:**
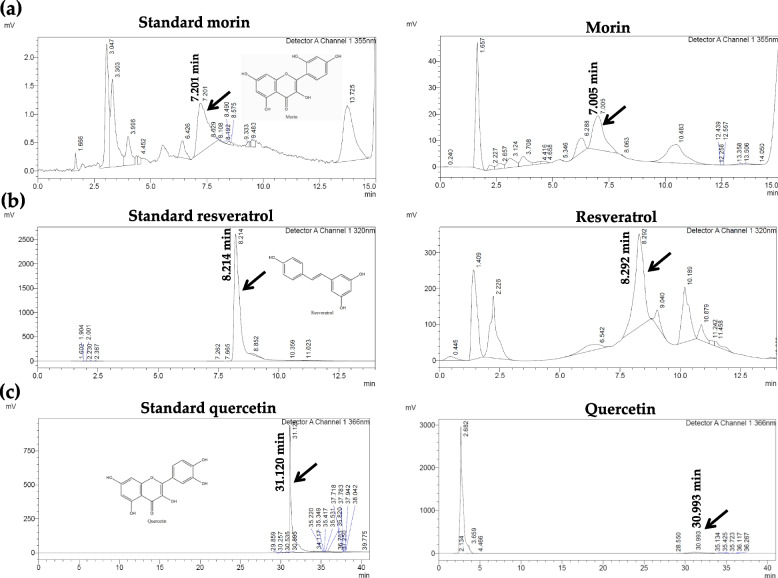
HPLC chromatograms of morin, resveratrol, and quercetin from EtOAc fractional extract No. 001. **a** Morin peak (7.0 min), **b** resveratrol (8.2 min), and **c** quercetin (31.0 min) were detected in EtOAc No. 001

**Fig. 2:**
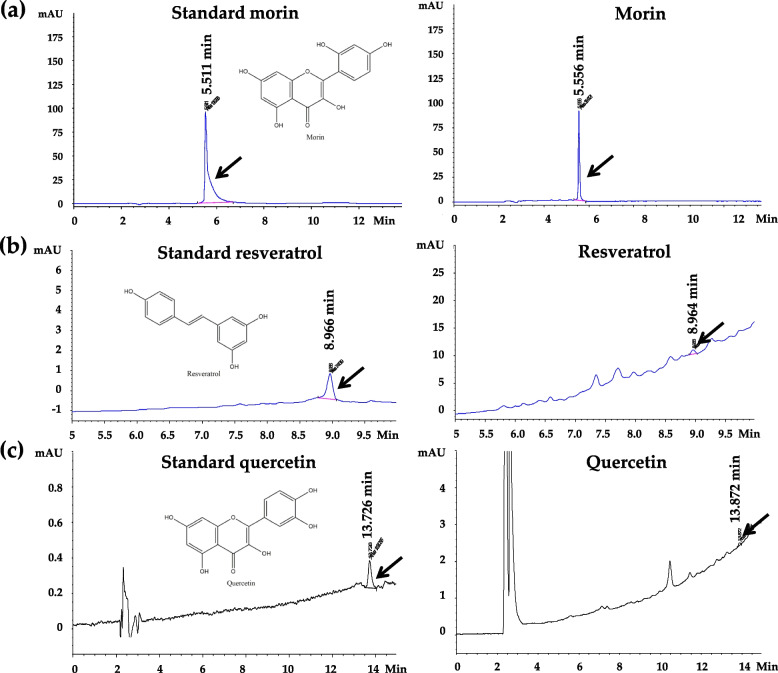
HPLC chromatograms of morin, resveratrol, and quercetin from Kae-Lae Hex No. 008. **a** Morin peak (5.5 min), **b** resveratrol (8.9 min), and **c** quercetin (13.7 min) were detected in Kae-Lae Hex No. 008

The morin, resveratrol, and quercetin contents of EtOAc No. 001 were 750, 6.032, and 147 g/kg, respectively. The results indicated that EtOAc No. 001 contained three compounds. Morin showed the highest content in EtOAc No. 001 (Fig. 1a), followed by quercetin (Fig. 1c) and resveratrol (Fig. 1b), respectively. Meanwhile, morin, resveratrol, and quercetin contents in Hex No. 008 were 334, 0.42, and < 0.01 mg/kg, respectively. Morin showed the highest content in Hex No. 008 (Fig. 2a), followed by resveratrol (Fig. 2b) and quercetin (Fig. 2c). The results indicated that both EtOAc No. 001 and Hex No. 008 contained three compounds.

### Cytotoxicity of morin, resveratrol, and quercetin in K562, KG-1a, EoL-1, and PBMCs by MTT assay

The cytotoxicity of pure compounds, morin, resveratrol, and quercetin in leukaemic cell lines were studied in three independent experiments using MTT assay. Table 3 shows that EoL-1 cell line was sensitive to three active compounds when compared with K562 and KG-1a cells. Morin had no cytotoxicity in both K562 and KG-1a cells with IC_50_ values > 100 µg/mL (Table 3). In contrast, resveratrol showed cytotoxicity in K562, EoL-1, and KG-1a with IC_50_ values of 41.63 ± 1.92, 5,49 ± 0.29, and 20.70 ± 2.65 µg/mL, respectively. Moreover, it also showed cytotoxicity in normal PBMCs with an IC_50_ value of 44.05 ± 9.70 µg/mL. Quercetin had cytotoxicity in EoL-1 and KG-1a cells.

**Table 3:** IC_50_ values (μg/mL) of morin, resveratrol, and quercetin in K562, KG-1a, EoL-1, and PBMCs

Compounds	IC_50_ values (mean ± SD, μg/mL)			
	K562	EoL-1	KG-1a	PBMCs
Morin	> 100	18.36 ± 1.60	> 100	> 100
Resveratrol	41.63 ± 1.92	5.49 ± 0.29^*^	20.70 ± 2.65^#^	44.05 ± 9.70
Quercetin	> 100	5.89 ± 0.46^*^	72.60 ± 7.45	> 100

Results expressed as mean ± SD of three independent experiments (*n* = 3). Asterisks (*) denote significant differences from morin (*p* < 0.05). Asterisks (^#^) denote significant differences from quercetin (*p* < 0.05)

### Antioxidant activity of crude fractional extracts and pure compounds

**Fig. 3:**
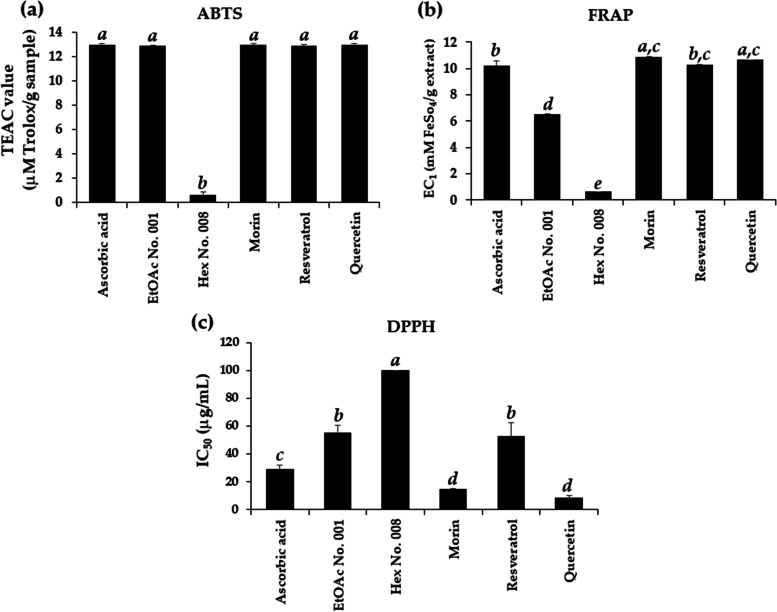
Antioxidant activities of crude fractional extracts and pure compounds from Kae-Lae. **a** ABTS, **b** FRAP, and **c** DPPH were examined for antioxidant activities. Results expressed as mean ± SD of triplicate sample (*n* = 3). The letters a-e denote significant differences among fractional extracts (EtOAc No. 001 and Hex No. 008) and dominant compounds (morin, resveratrol, and quercetin). The data were analysed using one-way ANOVA followed by post hoc Tukey’s test (*p* < 0.05)

The antioxidant activities of crude fractional extracts and their biological pure compounds, evaluated using ABTS, DPPH, and FRAP assays, are displayed in Fig. 3. EtOAc No. 001 showed significantly higher antioxidant activities than Hex No. 008 in all assays. Their biological pure compounds, including morin, resveratrol, and quercetin, had potent antioxidant activities and thus were suggested to be the bioactive compounds responsible for the antioxidant activities of EtOAc No. 001. Morin, resveratrol, and quercetin exhibited comparable TEAC and EC_1_ values with those of ascorbic acid, a well-known potent antioxidant. Interestingly, morin and quercetin were significantly more potent than ascorbic acid in DPPH^•^ inhibition. Therefore, it could be suggested that EtOAc No. 001 is an attractive natural extract with abundant natural antioxidants, especially morin, resveratrol, and quercetin.

### Effects of crude fractional extracts and pure compounds on anti-inflammatory activity

**Fig. 4:**
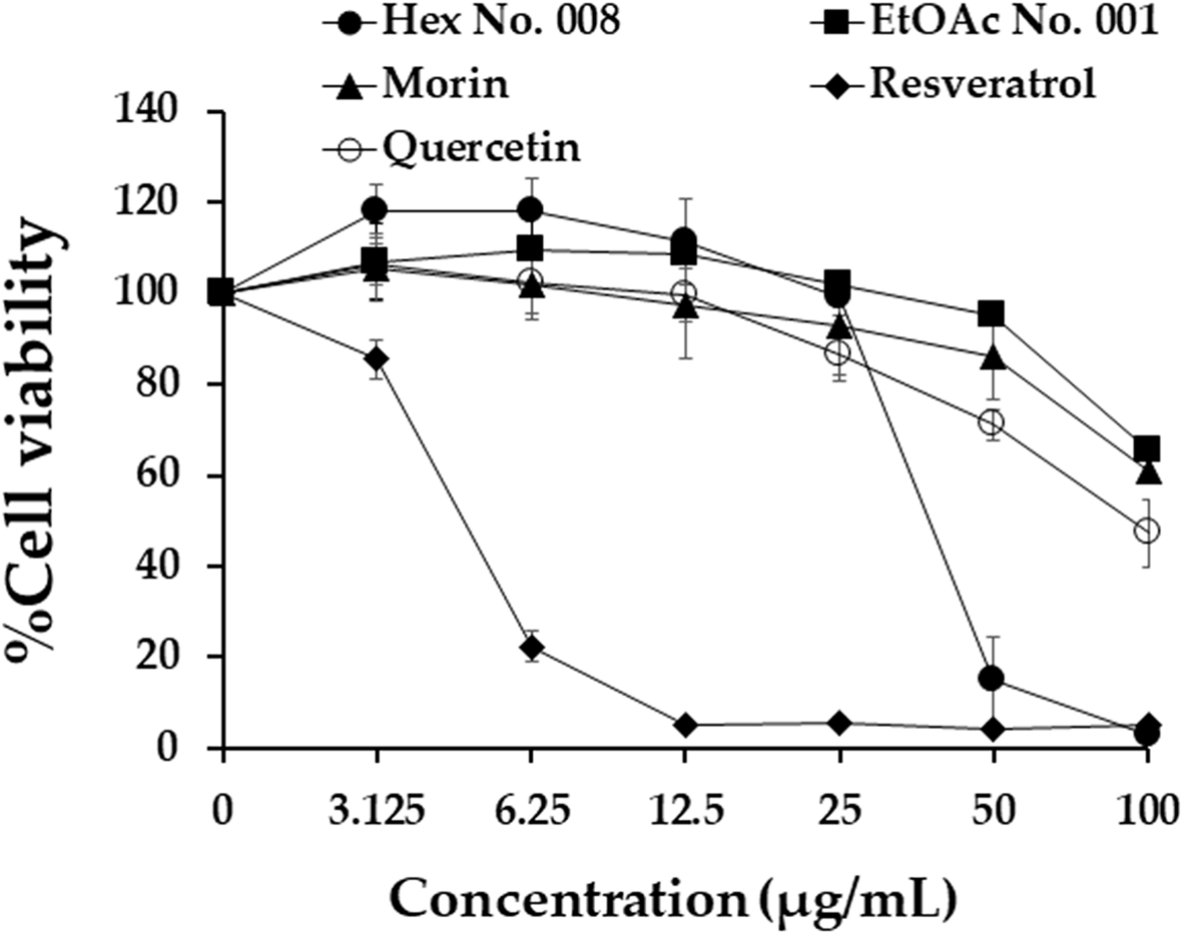
The effect of EtOAc No. 001, Hex No. 008, morin, resveratrol, and quercetin on the viability of RAW 264.7 cells using MTT assay. Each point represents the mean ± SD of three independent experiments performed in triplicate

**Fig. 5:**
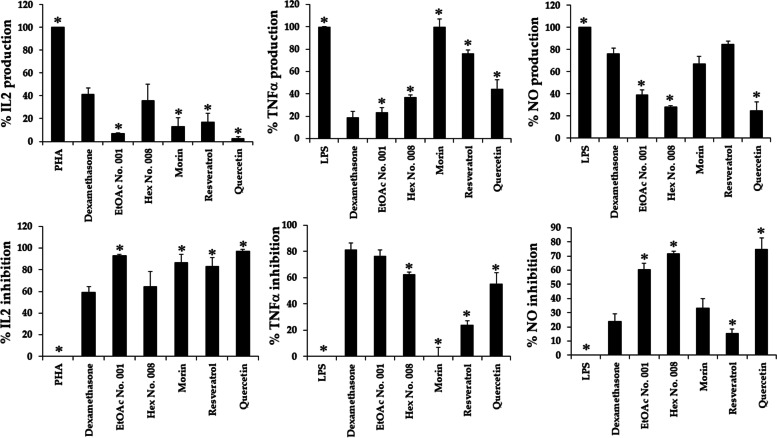
Anti-inflammatory activity (IL-2, TNF-α, and NO) of crude fractional extracts and pure compounds from Kae-Lae. Results expressed as mean ± SD of triplicate sample (*n* = 3). Asterisks (*) denote significant differences between Kae-Lae crude fractional extracts and active compounds (morin, resveratrol, and quercetin), and positive control (* *p* < 0.05)

**Fig. 6:**
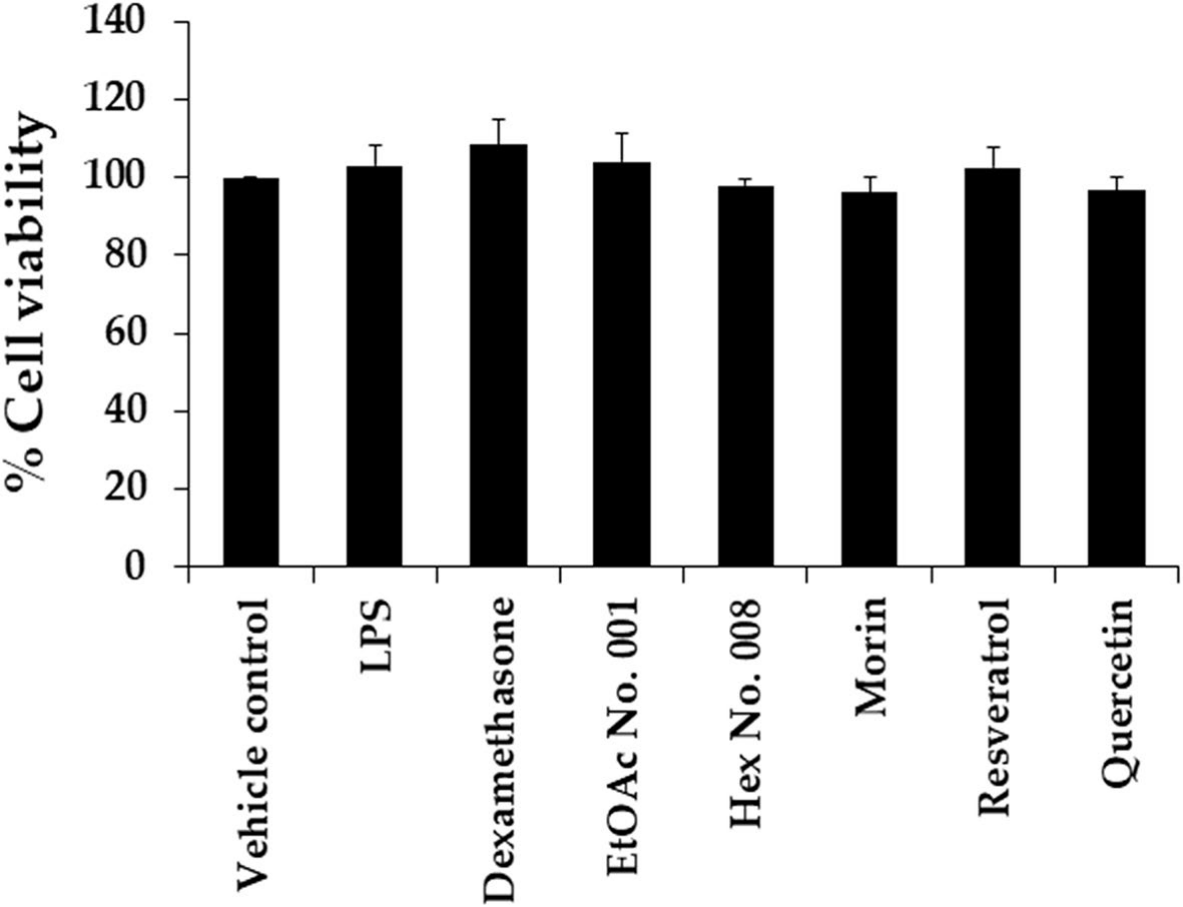
Effects of EtOAc No. 001, Hex No. 008, morin, resveratrol, and quercetin on RAW 264.7 cell viabilities after TNF- and NO determinations. Results expressed as mean ± SD of triplicate sample (*n* = 3)

The dose–response curves of RAW 264.7 cell viabilities following treatments with fractional extracts and three pure compounds are shown in Fig. 4. The IC_20_ values (concentrations at 80% of RAW 264.7 cells) of EtOAc No. 001, Hex No. 008, morin, resveratrol, and quercetin were 75.67 ± 2.59, 30.69 ± 1.02, 62.38 ± 7.44, 3.4 ± 0.20, and 35.52 ± 6.49 μg/mL, respectively. Therefore, EtOAc No. 001, morin, and quercetin tended to be safer for use in RAW 264.7 cells than Hex No. 008 and resveratrol. The concentration at the IC_20_ value of each sample was used for further anti-inflammatory activity determination. IL-2, TNF-α, and NO were used as markers for cancer prevention. Figure 5 illustrates the anti-inflammatory activity of both crude fractional extracts (EtOAc No. 001 and Hex No. 008) and their pure compounds (morin, resveratrol, and quercetin). When compared with the positive control (dexamethasone), EtOAc No. 001, morin, resveratrol, and quercetin suppressed IL-2 significantly better than dexamethasone. EtOAc No. 001, Hex No. 008, and quercetin showed potent inhibitory activities against NO (*p* < 0.05). Interestingly, EtOAc No. 001 and quercetin exhibited a significantly more potent inhibition of IL-2, TNF-α, and NO than dexamethasone. EtOAc No. 001 and Hex No. 008 exhibited TNF-α suppression in parallel with dexamethasone. Figure 6 shows that fractional extracts and their pure compounds at IC_20_ values did not affect RAW 264.7 cell viability. Therefore, decreasing TNF-α, and NO did not occur from decreasing of cell viabilities but from inhibiting TNF-α and NO productions after treatments.

### Effects of crude fractional extracts and pure compounds on normal red blood cell (RBC) haemolysis

To confirm non-cytotoxicity in normal cells, Kae-Lae fractional extracts (EtOAc No. 001 and Hex 008) and pure compounds were tested in normal RBCs. It was found that all Kae-Lae crude fractional extracts and pure compounds demonstrated a minimal effect on RBC haemolysis with a haemolysis rate comparable with that of the baseline haemolysis group, as shown in Table 4. Thus, all Kae-Lae crude fractional extracts and active compounds did not induce RBC haemolysis in vitro.

**Table 4:** Percentage of red blood cell haemolysis after treatments with EtOAc No. 001, Hex No. 008, morin, resveratrol, and quercetin

Samples	Concentrations (μg/mL)	Haemolysis (%)
0.05% Triton X100 (positive control)		100
0.9% Normal saline solution (negative control)		0
EtOAc No. 001	10	10.10 ± 1.75 ^*^
	50L	9.37 ± 1.64 ^*^
	100	9.67 ± 0.50 ^*^
Hex No. 008	40	10.77 ± 1.72 ^*^
	50	10.30 ± 0.62 ^*^
	60	11.16 ± 1.36 ^*^
EtOAc No. 012	40	10.18 ± 1.07 ^*^
	50	10.23 ± 1.23 ^*^
	60	11.29 ± 1.27 ^*^
Morin	50	9.39 ± 1.34 ^*^
	100	9.82 ± 1.09 ^*^
	150	10.22 ± 1.00 ^*^
Resveratrol	10	8.96 ± 1.83 ^*^
	20	8.58 ± 0.67 ^*^
	40	8.95 ± 0.21 ^*^
Quercetin	10	8.51 ± 0.86 ^*^
	50	8.85 ± 0.21^*^
	100	8.74 ± 0.26 ^*^

Results expressed as mean ± SD of triplicate samples (*n* = 3). Asterisks (*) denote significant differences between Kae-Lae crude fractional extracts and active compounds (morin, resveratrol, and quercetin), and positive control (^*^ *p* < 0.05)

### Effects of crude fractional extracts and pure compounds on WT1 protein expression and total cell numbers in K562, EoL-1, and KG-1a cells

**Fig. 7:**
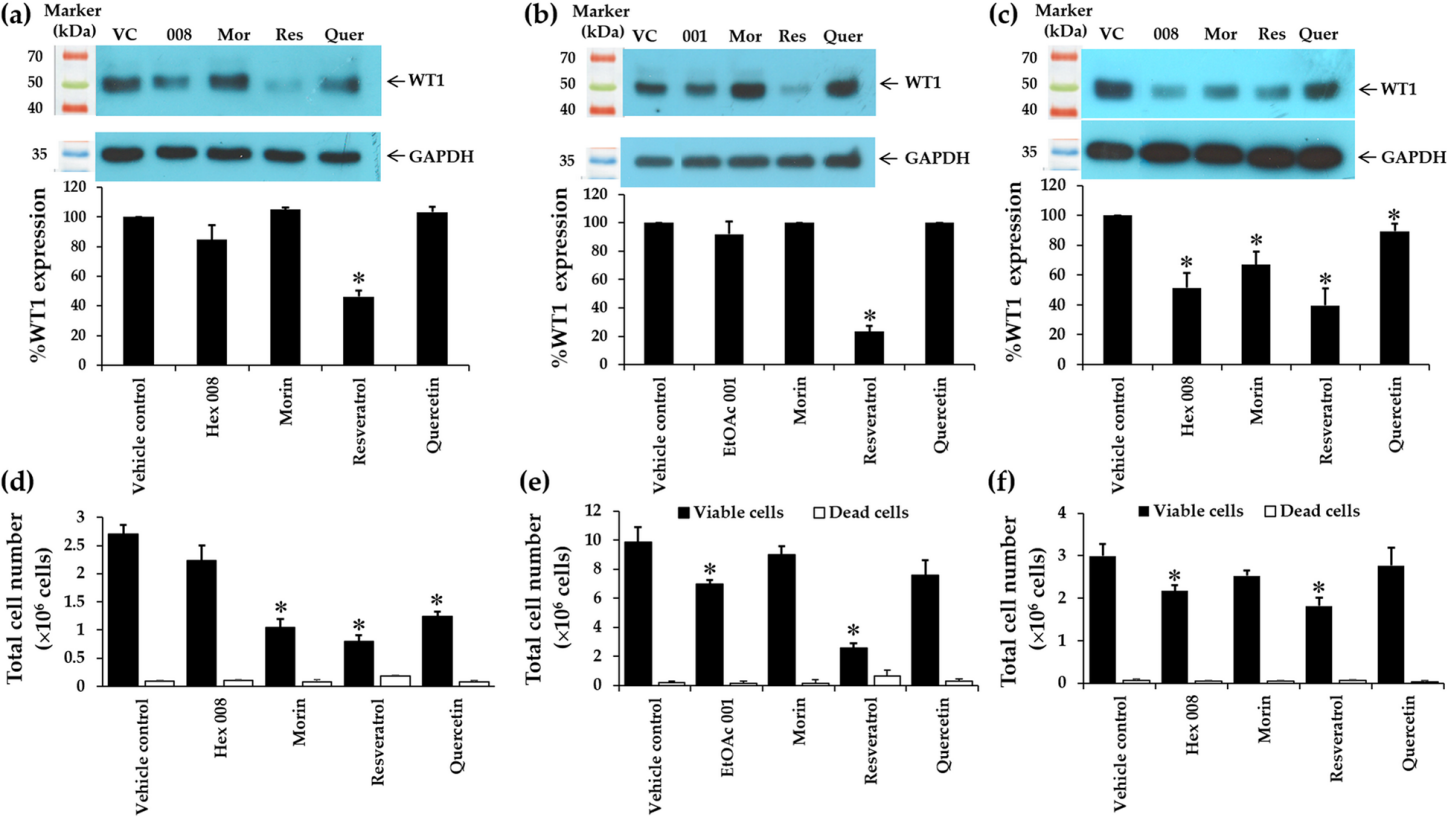
Effects of crude fractional extracts (EtOAC No. 001 and Hex No. 008) and pure compounds (morin, resveratrol, and quercetin) on WT1 expressions in K562, EoL-1, and KG-1a cell lines. **a** The level of WT1 protein in K562 cells following treatments with Hex No. 008 and pure compounds for 48 h. Protein levels were evaluated using Western blot and analysed using a scanning densitometer. The levels of WT1 were normalised using GAPDH protein levels. **b** The level of WT1 protein in EoL-1 cells following treatments with EtOAc No. 001 and active compounds for 48 h. **c** The level of WT1 protein in KG-1a cells following treatments with Hex No. 008 and pure compounds for 48 h. **d**, **e**, and **f** Total cell number of K562, EoL-1, and KG-1a following treatments with crude fractional extracts and pure compounds for 48 h. Total cell numbers were determined via the trypan blue exclusion method. Each bar represents mean ± SD of three independent experiments performed in triplicate. Asterisks (*) denote significant differences from the vehicle control (* *p* < 0.05)

Due to the effective cytotoxicity of EtOAc No. 001 (for EoL-1) and Hex No. 008 (for KG-1a and K562), inhibition of cell proliferation via WT1 protein expression was determined. The levels of WT1 protein in KG-1a cells after treatment with the Hex No. 008 and its pure compounds, including morin, resveratrol, and quercetin with IC_20_ values were detected by Western blotting. The results showed that resveratrol showed the maximum suppression of WT1 protein in K562, EoL-1, and KG-1a cells. It could significantly suppress WT1 protein expression by 53.78 ± 4.28, 76.73 ± 4.08, and 60.27 ± 11.44%, respectively (*p* < 0.05) when compared with the vehicle controls at 48 h (Fig. 7). However, Hex No. 008 and morin could also significantly suppress WT1 protein expressions in KG-1a cells by 48.47 ± 10.06 (*p* < 0.05) and 32.89 ± 8.76% (*p* < 0.05), respectively, when compared with the vehicle controls. In addition, the total cell numbers of EoL-1 cells after treatments with EtOAc No. 001 and resveratrol were significantly decreased by 29.26 and 74.14%, respectively when compared with the vehicle control (Fig. 7). Moreover, the total cell numbers of KG-1a cells after treatments with Hex No. 008 and resveratrol were significantly decreased by 27.25 and 39.23%, respectively (Fig. 7). All samples exhibited viable cells higher than 80% of the total cell count. Dead cells were less than 14.33 and 3.44% in EoL-1 and KG-1a cells, respectively.

### Effects of crude fractional extracts and pure compounds on rate of proliferation in K562, EoL-1, and KG-1a cell lines

**Fig. 8:**
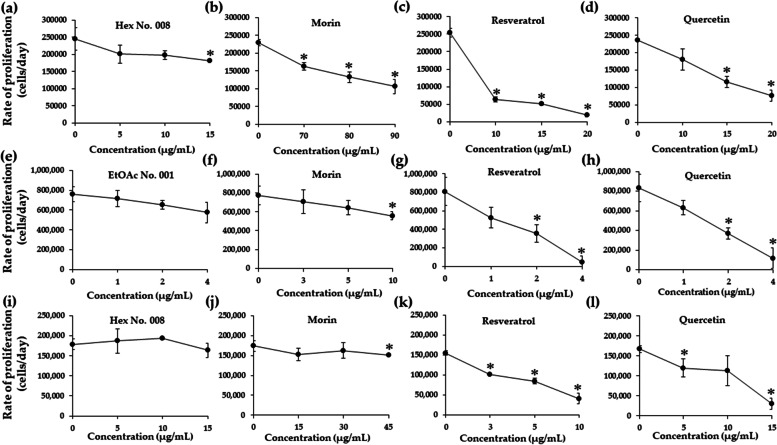
Effects of concentrations on the proliferation rate of K562 **a** − **d**, EoL-1 **e** − **h**, and KG-1a **i** − **l** cell lines cultured in fractional extracts and pure compounds. Following incubation of cells with various concentrations (IC_10_ − IC_20_ values) of fractional extracts and pure compounds, the number of leukaemic cells was determined after 48 h via trypan blue exclusion method. The slopes or cell growth rates at each predetermined fractional extract or pure compound concentration were determined from the plot of concentrations and cell numbers. Consequently, the rates of cell growth at different fractional extract or pure compound concentrations were compared with the normal growth rate, which was obtained from the control well (no compound). Each bar represents mean ± SD of three independent experiments performed in triplicate. Asterisk denotes significant differences from vehicle control; *p < 0.05

WT1 was reported to affect cell proliferation in leukaemic cells. Suppression of WT1 expression leads to decrease leukaemic cell numbers in K562, EoL-1, and KG-1a cells. To confirm total cell numbers after treatments, rates of cell proliferation were determined using trypan blue exclusion method. Resveratrol and quercetin showed a significantly decreasing rate of cell proliferation at concentrations ranging from IC_10_ − IC_20_ values for 48 h (Fig. 8c, d, g, h, k, l) when compared with morin (Fig. 8b, f, j), EtOAc No. 001 (Fig. 8e), and Hex No. 008 (Fig. 8a, and i) in three leukaemic cell lines. However, EtOAc No. 001 and Hex No. 008 decreased the rate of cell proliferation in EoL-1 (Fig. 8e) and K562 (Fig. 8a) cell lines, respectively. Morin showed a significant difference at concentrations of 70, 80, and 90 µg/mL, with the highest suppression in K562 cells when compared with those of EoL-1 and KG-1a cells (Fig. 8b, f, j).

**Fig. 9:**
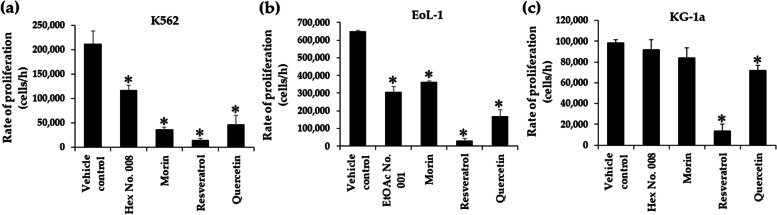
Effects of times on proliferation rate of K562 **a**, EoL-1 **b**, and KG-1a **c** cell lines cultured in fractional extracts and pure compounds. Following incubation of cells with the concentrations at IC_20_ values of fractional extracts and pure compounds, the number of leukaemic cells was determined after 24 and 48 h via trypan blue exclusion method. The slopes or cell growth rates at each predetermined fractional extract or pure compound concentration were determined from the plot of concentrations and cell numbers. Consequently, the rates of cell growth at different fractional extract or pure compound concentrations were compared with the normal growth rate, which was obtained from the control well (no compound). Each bar represents mean ± SD of three independent experiments performed in triplicate. Asterisk denotes significant differences from vehicle control; *p < 0.05

Effects of times (24 and 48 h) on rate proliferation showed a parallel pattern to the concentration treatments. The result showed that resveratrol and quercetin exhibited significantly decreasing rates of proliferation, more than morin in K562, EoL-1, and KG-1a cells. Furthermore, resveratrol showed a maximum decrease of rate proliferations at 93.20, 95.45, and 86.02% in K562, EoL-1, and KG-1a cells, respectively (Fig. 9a, b, and c). However, morin could also decrease the proliferation rate in K562 and EoL-1 cells. This study revealed that KG-1a cell line tolerates the effect of resveratrol, quercetin, and morin when compared with K562 and EoL-1 cell lines due to its leukaemic stem cell property. However, both resveratrol and quercetin can suppress rate proliferation in KG-1a cells (Fig. 9c).

### Effects of crude fractional extracts and pure compounds on cell migration

**Fig. 10:**
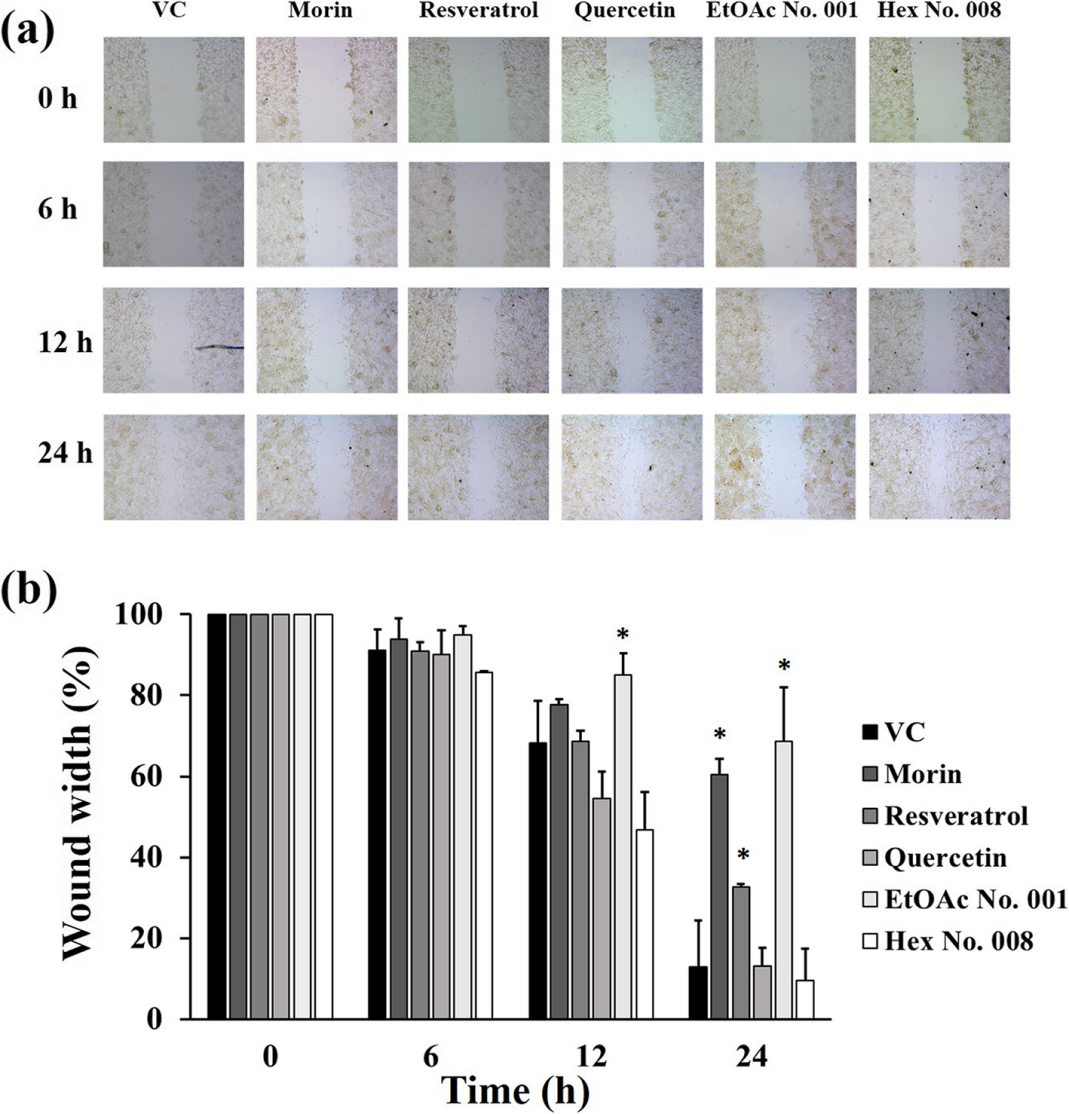
Effects of extracts on cell migration of MCF-7 cells. **a** Representative images of the wound area of MCF-7 cells after treatment with non-toxic concentration of morin, resveratrol, quercetin, fraction 1, and fraction 8 for 0, 6, 12, and 24 h compared with untreated group (vehicle control; VC). **b** Percentage of wound width was calculated according to the cell-free area of each treatment. The data are mean ± SD of three different areas. Asterisks denote statistical significance at * *p* < 0.05 versus VC of each time point

Inhibition of cell migration is the method used to determine cancer cell activity and function after compound or drug treatment. Normally, adherence cell line is always used as an experimental cell model due to its easy observation. In this study, MCF-7 breast cancer cell line was used as cell model. Our results showed that EtOAc No. 001, morin, and resveratrol showed inhibitory effects on MCF-7 cell migration when compared with the untreated group (vehicle control; VC) with a wound width of 68.6 ± 13.4, 60.5 ± 3.8, and 32.8 ± 0.7%, respectively, after 24 h of wounding (Fig. 10).

## Discussion

Kae-Lae (*M. cochinchinensis* (Lour.) Corner) is a medicinal plant for drug recipes in traditional Thai medicine. In this study, this plant extract was examined its biological activities, including antioxidant, anti-inflammatory, and anti-leukaemic activities. Crude fractional extracts obtained from 12 sources (in 9 provinces) in Thailand showed various cytotoxic values from different sources of plant materials and three leukaemic cell lines (K562, EoL-1, and KG-1a). Hex No. 008 from Phuket province and Hex No. 012 from Chiang Rai province revealed good activities in three leukaemic cell lines. Hex No. 008 was chosen for our further experimentation due to its higher yield. Meanwhile, EtOAc No. 001 from Bangkok province showed the best activity, especially in EoL-1 cell line with the IC_50_ value of 10.20 ± 2.08 μg/mL. However, it did not show significant difference from Hex No. 008 (12.21 ± 1.72 µg/mL). Moreover, EtOAc No. 001 showed a high percent yield. The variation of IC_50_ values is due to a variation of active compounds in the crude fractional extract in each area where the plants were collected. The reason may come from the nature of soil and agro-climatic conditions [3, 43]. Southern Thailand is suitable for productions of all active compounds in the Kae-Lae plant. When comparing the cytotoxicity between KG-1a cells and PBMCs, these extracts had stronger toxicity in normal cells than KG-1a cells. Thus, the concentration of the Hex No. 008 for further investigation of protein expression in KG1a cells was at IC_20_ value of PBMCs (Table 2). When comparing the cytotoxicity of plant extracts from Kae-Lae among K562, EoL-1, and KG1a cells, the results showed the highest cytotoxicities in EoL-1 cells versus KG1a and K562 cells due to the leukaemic stem cell property of KG-1a cells and the difference of the leukaemia type of K562 cells. K562 cells represent a chronic leukaemic cell type but EoL-1 cell is an acute leukaemic cell type. Thus, EtOAc No. 001 is suitable for AML treatment, while Hex No. 008 is suitable for CML, AML, and leukaemic stem cell treatments.

Compounds from the heartwood of Kae-Lae have been reported, such as resveratrol, oxyresveratrol, β-sitosterol, quercetin-7-O-β-D-glucoside, and kaempferol 3,7-di-O-β-glucopyranoside [2]. Among these compounds, morin was found predominantly in Kae-Lae heartwood. Thus, morin, resveratrol, and quercetin were studied to prove their biological activities when compared with EtOAc No. 001 and Hex No. 008. Morin was determined as the main compound in Hex No. 008 after HPLC determination (334 mg/kg), followed by resveratrol (0.42 mg/kg), and quercetin (< 0.01 mg/kg). This result was in parallel with the previous report [2]. Morin, resveratrol, and quercetin were suggested as antioxidants with potent radical-scavenging properties and ferric-reducing antioxidant powers. Comparing antioxidant activities of three pure compounds, it was found that morin, resveratrol, and quercetin exhibited TEAC and EC_1_ values comparable with those of standard ascorbic acid. Interestingly, morin and quercetin were significantly more potent than an ascorbic acid in the DPPH^•^ inhibition. EtOAc No. 001 showed significantly higher antioxidant activities than Hex No. 008 in all assays. Their biological pure compounds, including morin, resveratrol, and quercetin, had potent antioxidant activities and thus suggested being the bioactive compounds responsible for the antioxidant activities of EtOAc No. 001. All compounds contribute to the overall antioxidant activity of Kae-Lae extract. Since antioxidants have the ability to reduce oxidative stress in cells, they are useful for the treatment of various conditions, such as cancer, cardiovascular diseases, gastrointestinal diseases, inflammation, and neurodegenerative diseases [44–46]. Compound-092 was reported to decrease GSH levels but did not significantly increase intracellular ROS [47]. From previous studies, it was found that morin and resveratrol in Kae-Lae heartwood possess several biological activities, such as being an antioxidant, anti-inflammatory, etc. [48] and showed the effects on proliferation of leukaemic cells [27–34]. Moreover, the anti-haemolytic activity of Kae-Lae crude fractional extracts and active compounds might be related to the prevention of oxidative damage to the erythrocyte membrane owing to the presence of some antioxidants in the extracts [49]. Thus, it is safe for application in humans.

Anti-inflammatory research benefits the reduction of cancer incidence as a cancer prevention. Chronic inflammation can promote malignant transformation of cells and carcinogenesis. Diet plays a vital role in cancer management and prevention because it is the source of important physiologically functional components and nutrients. EtOAc No. 001 and Hex No. 008 showed excellent cancer prevention properties by inhibiting TNFα and NO production when compared with a positive control (dexamethasone). However, EtOAc No. 001 and quercetin exhibited a significantly more potent inhibition of IL-2, TNF-α, and NO than dexamethasone. It is possible that EtOAc No. 001 contained greater quercetin than resveratrol with a concentration of 147 g/kg. EtOAc No. 001 and Hex No. 008 exhibited TNF-α suppression in parallel with dexamethasone. Nitric oxide (NO) is an important molecule of the inflammatory cascade. Cordycepin was reported to inhibit ERK/Slug signaling activity in the A549 cell line. It revealed inhibition of LPS-induced inflammatory microenvironments, including TNFα, CCL5, IL-1β, IL-6, IL-8, NO, phosphor-ERK (p-ERK), and Slug expression [50]. Thus, it showed good benefits for cancer prevention. Moreover, previous studies revealed active compounds in fruits, vegetables, and spices, which had cancer protective effects. Sulforaphane and indole-3-carbinol in cruciferous vegetables [51–53] and lycopene in cooked tomatoes had cancer protective effects in humans. Previous studies indicated that high tomato consumption could reduce the risk of prostate cancer [54, 55].

TNF-α and IL-2 have been shown to participate in both initiation and progression of cancer. Moreover, pro-inflammatory cytokines also induce the generation of free radicals during chronic inflammation, such as inducible nitric oxide synthase (iNOS) and reactive oxygen species (ROS), which can damage DNA and cause mutations and tumor initiation [7]. Thus, inflammation has been demonstrated to play an important role in the initiation, progression, and prognosis of cancer. Thus, enhancing phagocytic activity of macrophages without stimulating inflammatory responses is essential in normal tissue homeostasis and in recovery from certain disease conditions, such as inflammation. Many previous studies have shown that there are compounds, including sulforaphane, lycopene, curcumin, resveratrol, and polyphenol [56–64], demonstrating cancer prevention properties by up-regulating phagocytic activity with inhibiting NO synthesis and cytokine release following LPS stimulation. Thus, the prevention of unregulated inflammation is one of the important methods for cancer prevention.

From the cytotoxicity of resveratrol on KG-1a cells, this result is related to the previous studies demonstrating that resveratrol sensitized leukemia stem cell-like KG-1a cells to cytokine-induced killer cells-mediated cytolysis through NKG2D ligands and TRAIL receptors [29]. Therefore, resveratrol may be an important active compound in the EtOAc and Hex fractional extracts of Kae-Lae. HPLC analysis showed that resveratrol was the compound in EtOAc No. 001 with a concentration of 6.032 g/kg, while morin was the main compound and the highest content in both EtOAc No. 001 (750 g/kg) and Hex No. 008 (334 mg/kg) when compared with those of resveratrol and quercetin.

Therefore, the EtOAc No. 001 and Hex No. 008 were selected for further investigation at their IC_20_ values of EoL-1 and KG-1a cells, respectively. They will be calculated and used as the concentrations for the determination of WT1 protein expressions in these cell lines. WT1 protein is a well-known biological marker of leukaemic cell proliferation [17–21]. The results were compared with morin, resveratrol, and quercetin at the concentrations of IC_20_ values. Overall results clearly exhibited that resveratrol showed the best activity to suppress WT1 protein expressions in EoL-1 and KG-1a cells when compared with morin and quercetin. Resveratrol could dominantly suppress WT1 protein expression with the highest value of 76.73% in EoL-1 cells, followed by KG-1a cells with the value of 60.28%. This is new knowledge about resveratrol on WT1 protein expression in leukaemic cells. According to WT1 protein involved in cell proliferation, suppression of WT1 can decrease the cell number in leukaemic cells. Thus, resveratrol showed the same inhibitory effect as the previous report of Mah-Lueang (*Curcuma* cf. *viridiflora* Roxb) [65]. The compounds (Germacrone-4,5-epoxide and zedoarondiol) from Mah-Lueang exhibited WT1 suppression activities against KG-1a leukaemic stem cells. Germacrone-4,5-epoxide and zedoarondiol decreased WT1 protein by 92.22 and 95.52% in KG-1a cells, respectively [65]. In our results, we found that quercetin showed good cytotoxicity in EoL-1 cells with an IC_50_ value of 5.89 ± 0.46 μg/mL. However, total cell number following quercetin treatment slightly decreased (23.25%). The reason comes from different methods and is to be determined. Cytotoxicity value comes from MTT assay, while the total cell number comes from trypan blue exclusion method. These two methods examine cell viability in different markers. MTT assay measures enzyme dehydrogenase from mitochondria but trypan blue exclusion measures cell permeability.

Moreover, Hex No. 008 had WT1 protein suppression activity and decreased total cell number in K562 and KG-1a cells. Meanwhile, EtOAc No. 001 slightly decreased WT1 protein expression (7.98%) in EoL-1 cells when compared with vehicle control. However, total cell number was significantly different between treatment and vehicle control with the value of 29.26% (*p* < 0.05). It is possible that other compounds in fractional crude extract in EtOAc could synergize to suppress cell proliferation in EoL-1 cells.

Following these results, rates of cell proliferation after EtOAc No. 001, Hex No. 008, morin, resveratrol, and quercetin were investigated to confirm the results of WT1 protein suppression related to the decreasing cell numbers. In this study, concentrations were designed for lower and higher IC_20_ values. The results showed that resveratrol trended to decrease the rates of cell proliferation in a dose-dependent manner. Moreover, quercetin also showed a decreasing rate of proliferation when increasing the concentration in both EoL-1 and KG-1a cells. EtOAc No. 001, Hex No. 008, and morin could slightly decrease the rates of cell proliferation in EoL-1 and KG-1a cells. As shown in the previous study, curcumin (5, 10, and 15 μM) from turmeric (*Curcuma longa* Linn) could decrease both *WT1* gene expression and then express a decreased rate of cell proliferation in K562, Molt4, U937, and HL-60 cells [66].

Cell migration was then tested to confirm the abilities of crude fractional extracts and pure compounds on cancer cell activity. Scratching technique by SPL™ Scar Scratcher was used to examine. Thus, leukaemic cells cannot be tested in this technique. Adherent cell line (MCF-7) was used as a cell model in this experiment. EtOAc No. 001, morin, and resveratrol showed good activity to inhibit cell migration in MCF-7 cells. Moreover, morin and resveratrol had higher content in EtOAc No. 001 than quercetin. Morin (150 − 200 µM) from leaves of *Cudrania tricuspidate* Bread suppressed cell migration by inhibiting the EGFR signaling pathway in SK-BR-3 cells [67]. Resveratrol could inhibit IGF-1-mediated cell migration of breast cancer MDA-MB 435 cells in vitro. The inhibitory effect was mediated in part through the suppression of the activation of PI-3 K/Akt signaling pathway [68].

## Conclusion

In this study, the fractional extracts from Kae-Lae were investigated for their biological activities, including antioxidant, anti-inflammatory, and anti-leukaemic properties. The results revealed that crude fractional extracts obtained from 12 sources of plant materials exhibited varying cytotoxic values against three leukaemic cell lines (K562, EoL-1, and KG-1a). Two crude fractional extracts (EtOAc No. 001 and Hex No. 008) and their dominant compounds (morin, resveratrol, and quercetin) demonstrated anti-leukaemic, antioxidant, anti-inflammatory activities, making them attractive natural extracts rich in natural antioxidants. Furthermore, these two crude fractional extracts were found to suppress WT1 protein expression, which is associated with leukaemic cell proliferation. Resveratrol, a potent antioxidant, anti-inflammatory, and cancer preventing agent, showed significant suppressions of WT1 protein expression, leukaemic cell proliferation, and cancer migration. Furthermore, resveratrol could decrease the rate of proliferation in leukaemic cells in a dose- and time-dependent manner. Interestingly, all compounds studied also exhibited cancer preventing activities that can protect normal cells from toxic substances in the environment, suggesting potential protective effects against human leukaemia, particularly acute myeloblastic leukaemia (AML), chronic myelocytic leukaemia (CML), and leukaemic stem cells (LSCs).

## Supplementary Information

**Additional file 1.** 

## Data Availability

The datasets used and/or analysed during the current study available from the corresponding author on reasonable request.
