# Supplementary material for: Biological activities of extracts and compounds from Thai Kae-Lae (Maclura cochinchinensis (Lour.) Corner)
Source: BMC Complement Med Ther. 2023 Jun 9;23:191. doi: 10.1186/s12906-023-03979-w (PMC10257291; doi:10.1186/s12906-023-03979-w)
Supplement: Supplementary file 1 — Additional file 1. [file 12906_2023_3979_MOESM1_ESM.pdf]

WT1 protein expression

K562

VC 008 Mor Res Quer

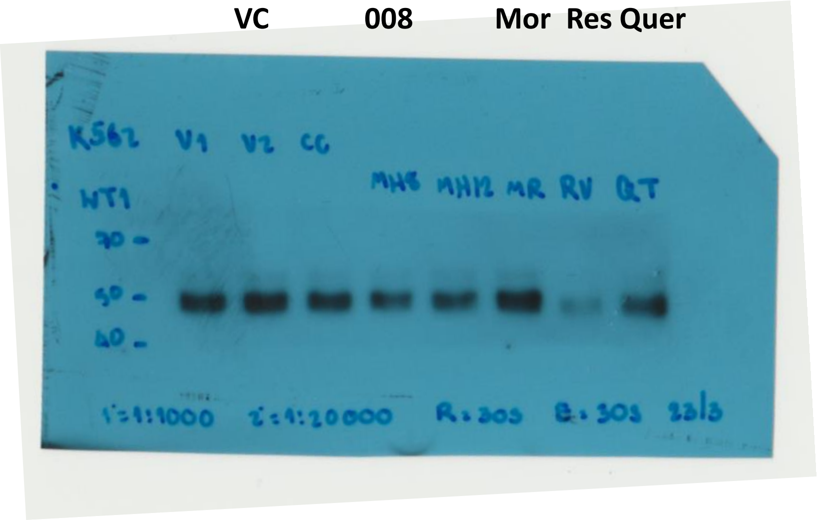

EoL-1

VC 001 Mor Res Quer

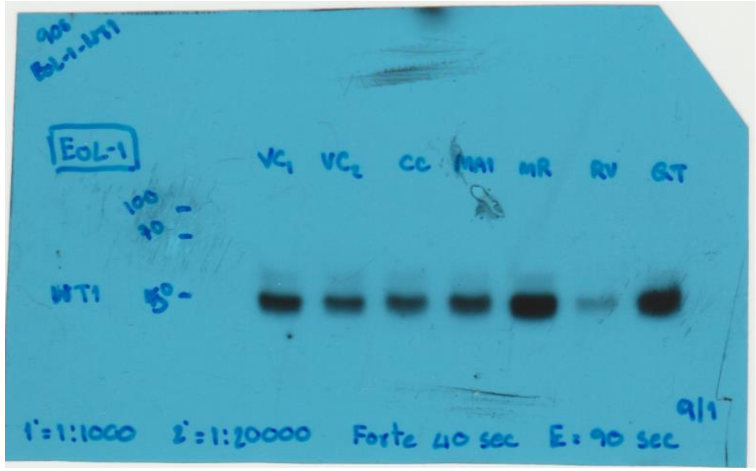

KG-1a

VC 008 Mor Res Quer

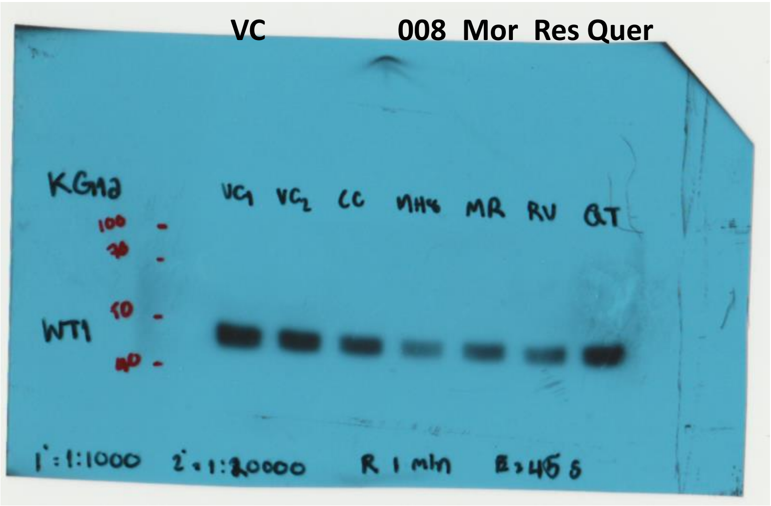

GAPDH protein expression

K562

VC 008 Mor Res Quer

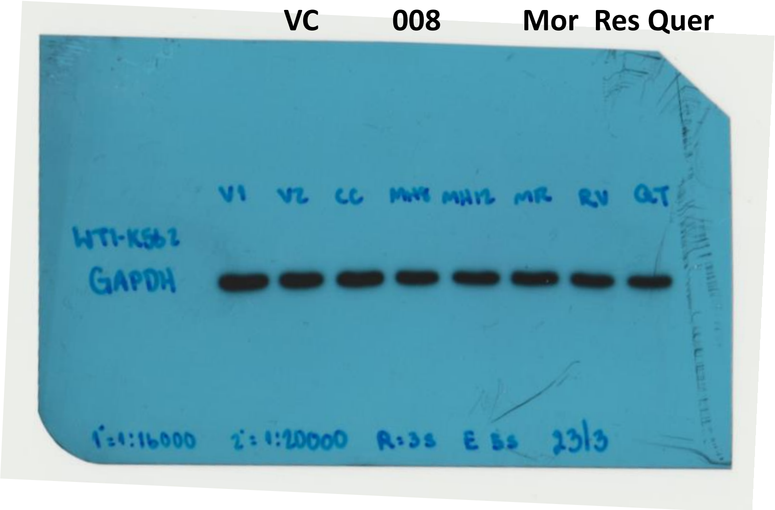

EoL-1

VC 001 Mor Res Quer

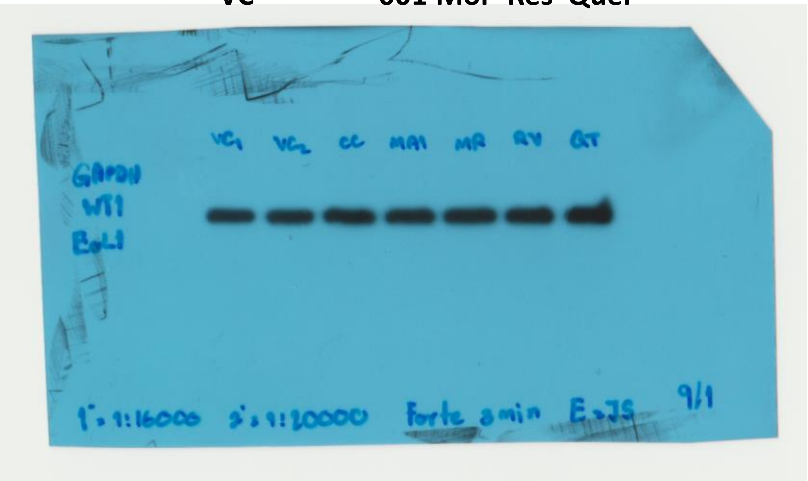

KG-1a

VC 008 Mor Res Quer

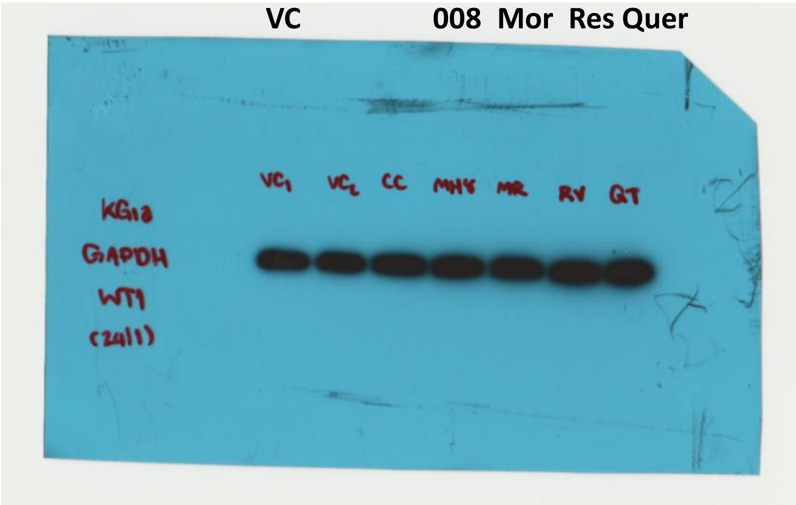

Labelling on X-ray film: V and VC = vehicle control, CC = Cell control, MH8 = *Maclura cochinchinensis* Hexane 008, MA8 = *Maclura cochinchinensis* Ethyl acetate 001, MR = Morin, Res = Resveratrol, QT = Quercetrin
